# A deep learning model for automated assessment of neonates at risk of impaired breast crawl: a proof-of-concept video-based study

**DOI:** 10.3389/fpubh.2026.1908206

**Published:** 2026-10-23

**Authors:** Zhu Xiang, Yunxuan Tong, Yuqi Chen, Anqi Xiong, Xinyu Shi, Biru Luo

**Affiliations:** 1 Department of Nursing, West China Second University Hospital, Sichuan University, Chengdu, Sichuan, China; 2 West China School of Nursing, Sichuan University, Chengdu, Sichuan, China; 3 Key Laboratory of Birth Defects and Related Diseases of Women and Children (Sichuan University), Ministry of Education, Chengdu, Sichuan, China

**Keywords:** breast crawl, deep learning, newborn, Shap, three-dimensional convolutional neural network (3D-CNN)

## Abstract

**Background:**

Timely initiation of breastfeeding is critical for newborn health. The breast crawl, an instinctive sequence of behaviors, is fundamental to this process. However, newborns vary considerably in their ability to complete the crawl, and current assessment relies on subjective endpoint observation. This approach cannot provide an early, objective measure of crawl competency or support timely intervention for at-risk mother-infant dyads.

**Objective:**

This study aimed to develop and validate a deep learning video analysis model for the automated, graded assessment of breast crawl competency and the identification of neonates at risk of impairment.

**Methods:**

In this prospective observational study, we recruited 114 full-term mother-newborn dyads from a tertiary hospital. Breast crawl sessions were video recorded, and 30 neonatal behaviors were annotated using an extended Widström framework. Because the model operates on the complete recorded episode (up to first suckling), it performs retrospective characterization of crawl competency rather than prospective prediction. We developed a two-stage framework. First, a three-dimensional convolutional neural network (3D-CNN) recognized 30 neonatal behaviors from multichannel video (RGB, pose, edge, and depth) to produce an action timeline. Second, 75 behavioral descriptors derived from this timeline were combined with 65 perinatal clinical variables in a fully connected network to predict a five-level competency grade. Shapley Additive Explanations (SHAP) was used to rank feature contributions.

**Results:**

Among 114 newborns (mean birth weight 3,185 g), the model showed moderate overall discriminative ability. It identified high-risk infants with F1-scores of 0.82 for Grade 3 (crawling without reaching the nipple) and 0.67 for Grade 4 (no crawling). Performance for the rare Grade 2 (reaching without attempting to suckle) was inconclusive because of the small sample (*n* = 3). On the held-out test set (*n* = 20), the model achieved a macro-average AUC of 0.77 and a micro-average AUC of 0.81. In a *post-hoc* binary analysis aligned with the intended clinical use (Grades 3–4 vs. 0–2), sensitivity was 1.00 (95% CI 0.69–1.00), specificity 0.70 (0.35–0.93), and accuracy 0.85 (0.62–0.97). Confidence intervals were wide throughout, reflecting the small test set. Grade 2 (*n* = 3) could not be evaluated. SHAP analysis identified an earlier onset of hand-to-perioral activity and a shorter interval between maternal pethidine administration and delivery as among the most salient features associated with better and poorer crawl competency, respectively.

**Conclusion:**

This proof-of-concept study suggests that deep learning video analysis may provide an objective adjunct for identifying neonates at risk of impaired breast crawl. However, the estimates are derived from a single center with a small test set and a non-nested feature selection procedure, and should be regarded as preliminary and hypothesis-generating. External validation with a prespecified nested analysis plan is required before any clinical application.

## 1 Introduction

The neonatal period (birth to 28 days) is the most vulnerable phase of life, and neonatal mortality remains a critical global health challenge. Deaths during this period account for 47% of all deaths among children under 5 years of age (1), and mortality is disproportionately concentrated among poor, rural, and vulnerable populations with limited access to quality care (2). In 2013, the World Health Organization (WHO) launched the Early Essential Newborn Care (EENC) guidelines to strengthen maternal and neonatal health services and reduce neonatal mortality. These guidelines were first implemented in eight Western Pacific countries with relatively high maternal and infant mortality rates, including China (3). Immediate and uninterrupted skin-to-skin contact (SSC) between mothers and newborns serves as a core intervention of the EENC guidelines. During SSC, the newborn independently crawls to the mother’s breast, locates the nipple, attaches, and begins to suck. This natural behavior is termed the breast crawl (BC) (4).

This process was first systematically described by Widström et al. in 1987 (4), and further refined it into nine distinct stages in 2011, with first suckling typically completed within 30–60 min of birth (5). The breast crawl is crucial for the success of early breastfeeding and also influences neonatal nutritional status and growth (6). Studies show that the breast crawl helps reduce neonatal mortality through sustained mother-infant contact (7, 8),and facilitates neonatal adaptation to the extrauterine environment, relieves neonatal stress and discomfort after birth, and prevents common neonatal complications such as hypothermia and hypoglycemia (8, 9). For postpartum women, the passive breast stimulation generated by neonatal crawling can effectively promote lactation and oxytocin secretion. This process facilitates complete placental delivery, reduces the risk of postpartum hemorrhage, and accelerates uterine involution within 24 h after delivery (10). It can also improve hemoglobin levels at discharge among mothers who delivered by cesarean section (10).

Although the clinical value of breast crawl is well acknowledged, large-scale implementation of this intervention remains limited in routine clinical care. This translational gap arises from two major practical barriers. On the one hand, breast crawl represents a complex, dynamic behavioral sequence with prominent individual variations in duration, efficiency, and final outcomes. Conventional manual real-time observation is time- and labor-consuming, susceptible to subjective bias, and difficult to standardize in clinical settings. On the other hand, current research mostly evaluates breast crawl outcomes through binary judgment criteria, such as successful breastfeeding within a fixed time window, or relies solely on single indicators like the time to first suckling. Such evaluation methods cannot fully reflect the multidimensional characteristics of breast crawl performance, including behavioral efficiency, movement coordination, and the overall rationality of behavioral sequence organization. Therefore, objective, refined, and automated assessment tools are urgently required to quantify breast crawl ability as a continuous graded indicator, rather than a simplistic binary endpoint.

Recent advances in artificial intelligence have offered viable solutions to overcome these limitations. Deep learning techniques, particularly convolutional neural networks (CNNs), have demonstrated outstanding performance in various computer vision tasks owing to their powerful capability for visual feature extraction (11). Three-dimensional CNNs (3D-CNNs) are specifically designed to capture integrated spatiotemporal features from consecutive video frames, making them highly suitable for analyzing continuous behavioral movements (12). Despite these advantages, the inherent “black-box” property of deep learning models hinders their widespread application in clinical scenarios. To mitigate this issue, explainable artificial intelligence (XAI) approaches such as SHAP allow researchers to interpret model predictions, identify key predictive features, and build trust by linking computational outputs to clinical understanding (13).

This study developed and validated a deep learning-based video analysis framework for the automated and graded evaluation of neonatal breast crawl competence. Integrated with SHAP interpretability analysis, the framework identified and prioritized key behavioral and clinical features that drive model predictions, clarifying the core determinants of neonatal breast crawl performance. Different from conventional descriptive observation methods, this novel tool enables quantitative, interpretable, and efficient assessment of breast crawl behavior. It not only advances the scientific comprehension of this innate neonatal behavior but also provides an objective, automated characterization of breast crawl performance. By analyzing full-length breast crawl video episodes, the model achieves behavioral characterization of crawl competency instead of prospective prediction. It can help clinicians objectively identify newborns with suboptimal crawling performance and develop individualized care plans, further improving breastfeeding outcomes and overall neonatal health.

## 2 Methods

### 2.1 Study design and participants

We conducted a prospective observational study at a tertiary women’s and children’s hospital in China from May to December 2023. The study involving human participants was reviewed and approved by the Ethics Committee of West China Second University Hospital, Sichuan University (Approval No. 2023076). The study was conducted in accordance with local legislation and institutional requirements. Written informed consent was obtained from all adult participants or, where applicable, from their legally authorized representatives.

Inclusion criteria were: (1) maternal age 20 years or older; (2) no communication barriers; (3) spontaneous vaginal delivery; and (4) gestational age between 37 and 42 weeks.

Exclusion criteria were: (1) medical contraindications to breastfeeding; (2) maternal unwillingness to breastfeed; (3) multiple gestation; (4) neonatal Apgar score below 8 at 5 min; and (5) major congenital anomalies.

Dyads were also excluded if the mother required urgent postpartum care, if mother and newborn were separated for more than 15 min, or if the newborn received assistance with areolar positioning before initiating the crawl. A total of 114 mother-newborn dyads were included in the final analysis.

### 2.2 Video recording of the breast crawl

Immediately after delivery, trained midwives facilitated skin-to-skin contact following the WHO EENC guidelines. Recording began at complete delivery of the newborn and continued until first nutritive suckling was completed. In line with the study protocol, no assistance was provided during the first 45 min (14). Thereafter, if a neonate had reached the areola but showed no rooting response or could not suckle spontaneously, the midwife gently stroked the neonate’s lips with the maternal nipple to elicit the rooting and sucking reflexes, so that feeding was not unduly delayed. Competency grade was assigned from behavior observed before any such assistance; the provision of assistance is recorded and discussed as a limitation.

A 720p HD video camera (HYUNDAI HY-8H) was positioned at the head of the delivery bed to record the entire breast crawl. Both video and audio data were collected. Because recording ended at first suckling, the observation window varied between dyads (mean 52.26 ± 15.77 min) and is itself related to competency. Behavioral frequency and duration features were therefore derived over windows of differing length; the implications are discussed in the Limitations section. The model takes complete videos recorded until the first suckling as input, enabling retrospective behavioral characterization of finished breast crawl episodes instead of prospective prediction based on incomplete early video segments.

### 2.3 Annotation of neonatal breast crawl behaviors

#### 2.3.1 Behavioral taxonomy

We developed an action classification tool based on the nine-stage breast crawl described by Widström et al. (5), supplemented by action categories and definitions from other studies and by additional actions observed in this study. Through repeated expert review and group discussion, we identified 30 distinct neonatal breast crawl behaviors.

#### 2.3.2 Annotation process

Nineteen of the 114 recordings (17%) were randomly selected for detailed action annotation. Two researchers trained in the behavioral taxonomy annotated these recordings jointly and resolved every disagreement through discussion until full consensus was reached on all action categories and timestamps. This consensus set of 12,176 action instances formed the ground truth for training the action recognition model. Annotation and competency grading of the remaining recordings were performed by a single trained researcher using the agreed criteria. Video segments affected by external disturbances, such as involuntary neonatal movements during ward transfers, were excluded from annotation. Labeling resumed immediately after interfering events ceased. All annotations were completed through collective consensus discussion rather than independent individual scoring, which eliminated redundant labeling data and precluded the calculation of inter-rater reliability. The corresponding limitations and implications of this annotation strategy are elaborated in the Limitations section of this study.

### 2.4 Definition and assessment of breast crawl competency

This study defined and quantified neonatal breast crawl competency based on preliminary experimental observations and professional expert consensus. Breast crawl performance was operationalized as a five-level ordinal grading system (Grades 0–4) that encompasses two core behavioral dimensions: spontaneous progression toward the maternal nipple, and successful nipple latching and subsequent suckling initiation. A higher grade indicates poorer autonomous breast crawl performance (Table 1).

**Table 1:** Breast crawl competency grading scale.

Grade	Meaning
0	Neonate autonomously crawls to nipple, latches, and achieves nutritive suckling
1	Neonate crawls to nipple and attempts latching/suckling but is unsuccessful
2	Neonate crawls to nipple but does not attempt latching/suckling
3	Crawling movements are observed, but neonate does not reach nipple autonomously
4	No crawling movements are observed.

### 2.5 Deep learning model development

#### 2.5.1 Overview of the two-stage framework

The proposed framework consists of two sequentially trained stages (Figure 1).

**Figure 1:**
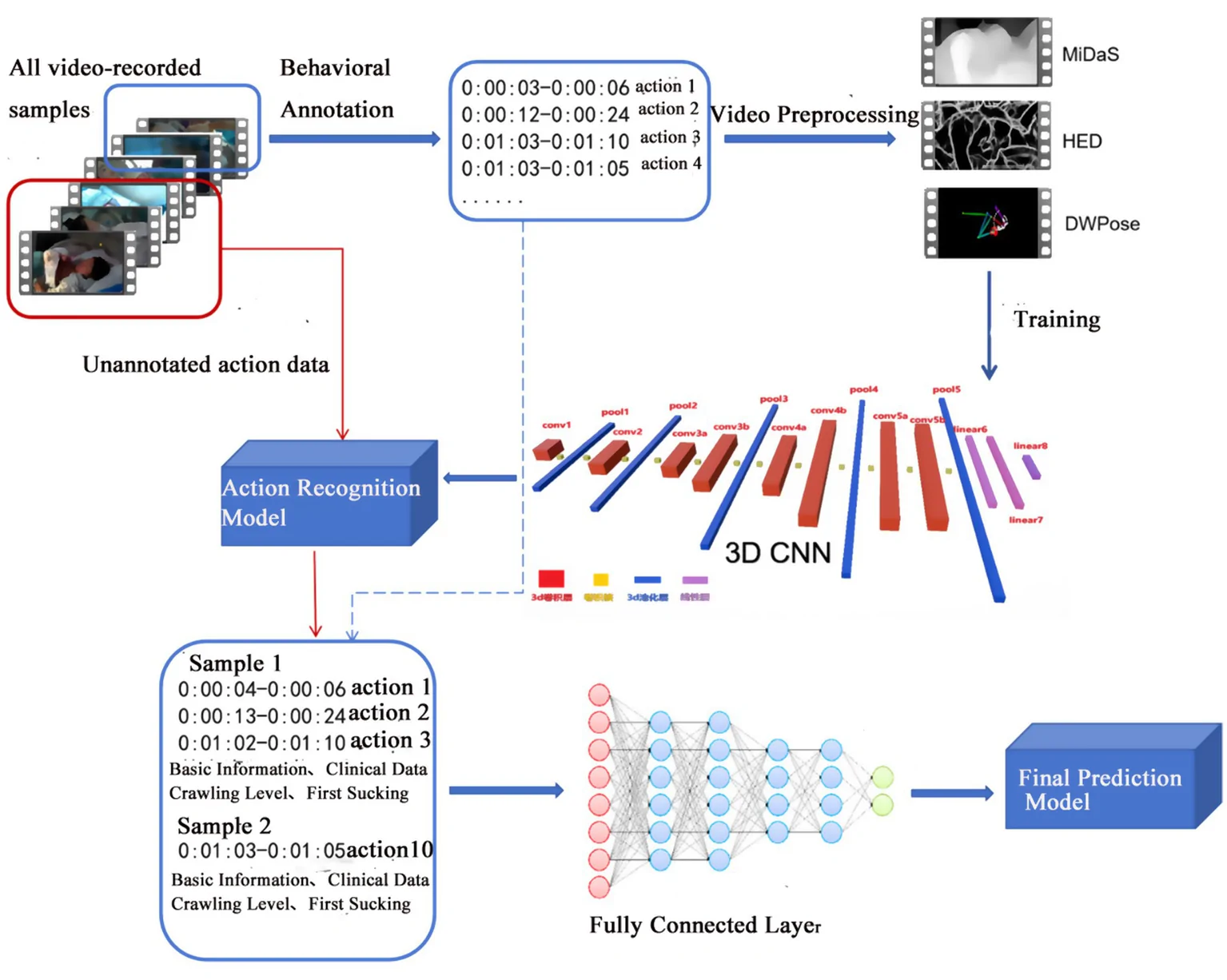
Development workflow of the predictive model.

In Stage 1, a three-dimensional convolutional neural network (3D-CNN) receives a short multichannel video clip and indicates, for each of the 30 predefined behaviors, whether that behavior is present in the clip. Applied in a multiscale sliding-window manner across the entire recording, Stage 1 converts raw video into an action timeline that records, for each behavior, the time of first occurrence, the number of occurrences, and the cumulative duration.

In Stage 2, quantitative descriptors derived from the action timeline are combined with maternal, neonatal, and obstetric variables and passed to a fully connected network that outputs the breast crawl competency grade.

The two stages use different labels. Stage 1 is trained on the frame-level action annotations described in Section 2.3, and Stage 2 on the dyad-level competency grades described in Section 2.4. SHAP analysis (Section 2.6) is applied to the Stage 2 network because this model maps clinically interpretable features onto the outcome of interest.

All models were implemented in PyTorch 2.2.1 with Python 3.10.12 on Ubuntu 22.04.3 LTS, and image processing used OpenCV (opencv-python 4.9.0.80). Training and inference ran on a single NVIDIA RTX 4090 GPU with an Intel Core i7-12700F CPU.

#### 2.5.2 Video preprocessing and multichannel feature extraction

Preprocessing followed the principle of knowledge distillation, in which large pretrained “teacher” models supply enriched representations to a compact task-specific network, allowing the latter to achieve comparable performance with fewer parameters and less training data (15). Neonatal breast crawl video contains several distinct types of information, including limb and trunk movement, the neonate’s position relative to the maternal body and the nipple-areola complex, and depth relationships within the scene. We therefore selected three complementary teacher models, each addressing one of these.

Whole-body pose was estimated with DWPose (16), a two-stage distillation method, to capture limb and trunk activity. Contour information was extracted with Holistically-Nested Edge Detection (HED) (17) to capture the neonate’s position relative to the maternal body and the location of the nipple and areola. This study adopted MiDaS (18) for relative monocular depth estimation to improve model adaptability to diverse camera distances and scenarios. All three models were used with frozen pretrained weights and were not fine-tuned on our data.

From each annotated behavioral segment, 12 frames were uniformly sampled at equal intervals. All frames were resized to a resolution of 288 × 512 pixels. This dimension was selected to balance visual clarity with GPU memory consumption and inference efficiency. Each resized frame was subsequently fed into the three teacher models. The generated single-channel pose, edge, and depth maps were concatenated with the original three RGB channels, forming a six-channel feature representation of (512, 288, 6). The 12 sampled frames were stacked chronologically to construct a 4D sample with the dimension of (12, 512, 288, 6). During model training, all samples were grouped into batches with a unified shape of (n, 12, 512, 288, 6).

#### 2.5.3 Stage 1: 3D-CNN for neonatal action recognition

(a) Network architecture.

The model backbone adopted a 3D convolutional neural network architecture proposed by Ji et al. (12). This network performs convolution across width, height, and temporal dimensions, extracting motion features from consecutive frame sequences instead of isolated single frames. The output layer contained 30 neurons, corresponding to the total number of behavioral categories. A dropout rate of 0.10 was implemented after each convolutional block to mitigate overfitting. Figure 2 visualizes the overall training and inference pipeline.

(b) Training.

**Figure 2:**
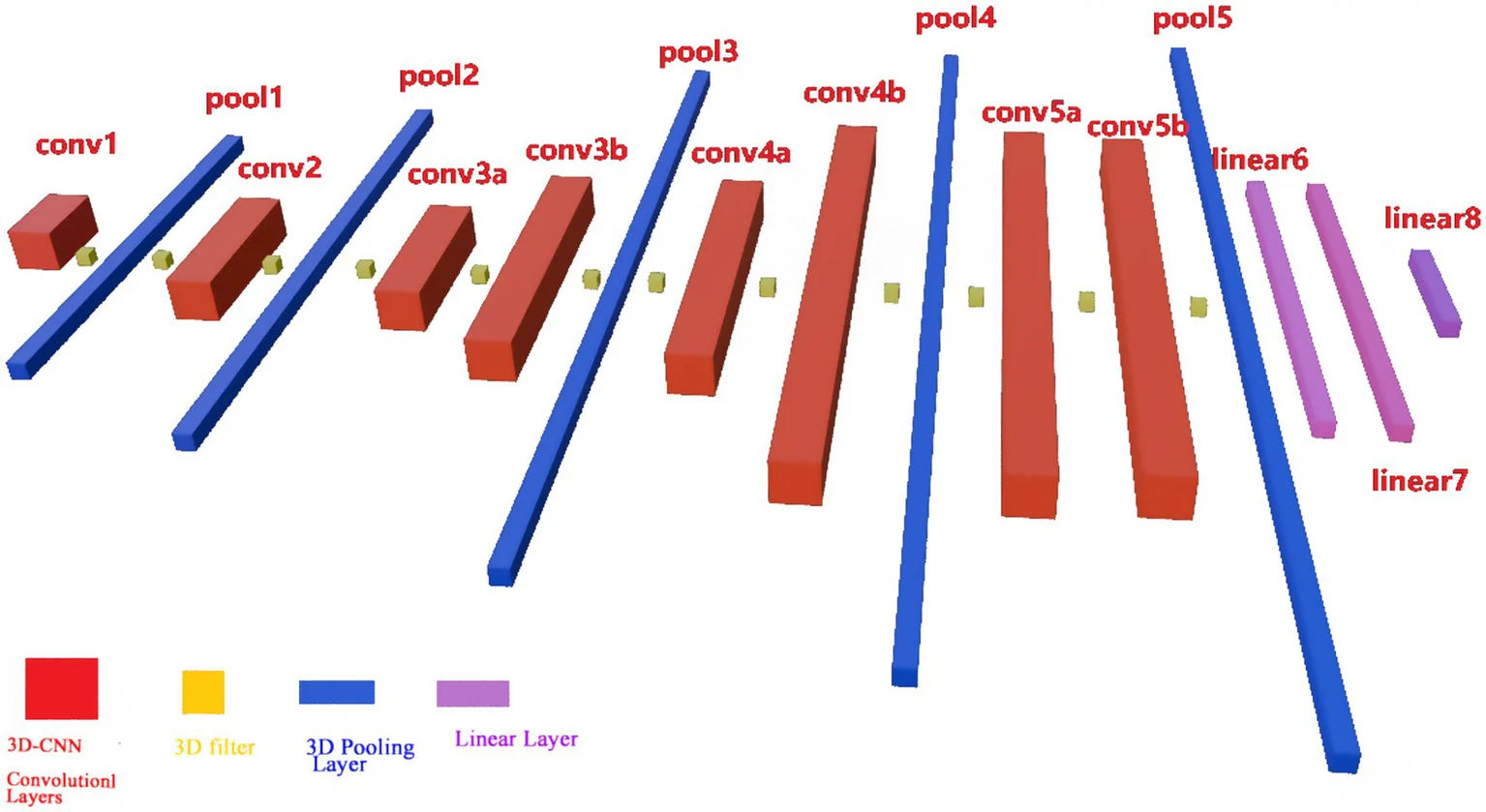
3D-CNN training flowchart.

Stage 1 model training was performed on manually annotated action instances described in Section 2.3.2. In total, 12,176 annotated samples from 19 video recordings were randomly split into training (70%, *n* = 8,777), validation (10%, *n* = 976), and test (20%, *n* = 2,423) subsets. The behavioral category distribution across subsets is summarized in Supplementary Table S2. Model optimization was implemented via stochastic gradient descent with a batch size of 1 and an initial learning rate of 1 × 10^−5^. The learning rate decayed by a factor of 10 every 10 epochs throughout the 103-epoch training process. Validation loss and accuracy were evaluated after each epoch to guide iterative hyperparameter tuning.

- (c) Inference: from video to action timeline.

Breast crawl behaviors vary substantially in temporal duration. Brief events such as eye opening and minor hand movements occur within a fraction of a second, while complete crawling and hand–breast–mouth behavioral sequences require far longer execution time. To address this temporal heterogeneity, we adopted six parallel temporal sampling scales, with window sampling rates set at 12, 10, 8, 6, 4, and 2 frames per window. Shorter windows effectively capture transient micro-behaviors, whereas longer windows accommodate sustained, extended behavioral sequences.

For each temporal scale, frames were aggregated to form fixed windows of 12 frames. Each qualified window was preprocessed following the procedures in Section 2.5.2 and subsequently fed into the trained 3D-CNN model. The network outputted a 30-dimensional vector that represents the occurrence probability of each behavioral category within the corresponding window. Behaviors detected across temporally adjacent windows were merged as a single continuous instance, with their total duration accumulated accordingly. By contrast, interrupted behaviors that reappeared after a pause were recognized as new independent occurrences. During the fusion of multi-scale outputs, redundant overlapping detections from the six temporal scales were eliminated to ensure detection accuracy.

Two behaviors were handled outside this pipeline. Relaxation and rest were not manually annotated because both are defined by the absence of movement rather than by a positive motor pattern; rest was derived algorithmically as any cessation of movement exceeding 15 s.

#### 2.5.4 Stage 2: competency prediction model

(a) Behavioral features.

For each behavior, three descriptors were considered: time of first occurrence, cumulative duration, and number of occurrences. Descriptors lacking clinical meaning were excluded before modeling, on the grounds that retaining them would add noise rather than information.

Duration was excluded for behaviors that are instantaneous by definition: correct latch and suckling, holding the nipple, lips touching the nipple or areola, tongue touching the nipple or areola, locating the breast or areola, hand-breast-mouth movement, mouthing and sucking movements, cooing, tongue protrusion, and eye opening. For eye opening and correct latch and suckling, only the time of first occurrence was retained, because neither frequency nor duration carries interpretable meaning for these events. Relaxation was excluded entirely.

This yielded 75 behavioral features. The complete specification of the descriptor retained for each behavior is given in Supplementary Table S3.

- (b) Clinical features.

Sixty-five maternal, neonatal, and obstetric variables were extracted from the hospital information system and included as candidate predictors; the complete list is given in Supplementary Table S4.

(c) Network and training.

The 140 candidate features (75 behavioral and 65 clinical) were passed to a fully connected network with five output units corresponding to the five competency grades (Supplementary Table S1). Dropout (rate 0.10) was applied between fully connected layers. Optimization used the Adam optimizer with a batch size of 1 and an initial learning rate of 1 × 10^−4^, reduced by a factor of ten every 30 epochs, for 1,000 epochs.

The number of candidate features exceeded the number of dyads available for training (*n* = 94). No prespecified dimensionality reduction was applied before modeling; feature reduction was instead performed *post hoc* using SHAP values, as described in Section 2.6. The implications are considered in the Limitations section.

### 2.6 Model interpretability: SHAP analysis

Deep neural networks of the kind used here are not directly interpretable, and interpretability is increasingly regarded as a prerequisite for clinical adoption. We therefore applied Shapley Additive Explanations (SHAP) (13), a method grounded in cooperative game theory that attributes a model’s prediction to its individual input features. For a given sample, the SHAP value of a feature quantifies that feature’s contribution to the predicted output relative to a baseline; a positive value indicates that the feature increased the predicted value, and a negative value that it decreased it. Analyses used the shap package (v0.45.0).

SHAP served two purposes. First, for feature reduction: after the initial model was fitted, the mean absolute SHAP value of each feature was computed across all samples and used to rank features by global importance. All 75 behavioral features were retained; clinical variables with a mean absolute SHAP value at or below 0.025 were discarded. The remaining 95 features were used to refit the network, and this reconstructed model is the one whose performance is reported in Section 3. Second, for interpretation: global importance was summarized by mean absolute SHAP value, and the direction of each feature’s effect was examined using SHAP summary plots, in which each point represents one sample’s SHAP value for one feature, positioned horizontally by SHAP value and colored by the magnitude of the underlying feature value.

This feature reduction used the complete dataset, including the cases subsequently used for testing, rather than being nested within the evaluation split. The implications for the reported performance are discussed in the Limitations section.

SHAP quantifies each feature’s contribution to the model’s output and does not establish causal relationships; the associations reported in Section 3.6 and interpreted in Section 4 should be read accordingly.

### 2.7 Performance evaluation metrics

We used three types of metrics. Classification metrics included accuracy, precision, recall (sensitivity), specificity, and F1-score. Composite metrics included the receiver operating characteristic (ROC) curve and area under the curve (AUC). Visualization included the confusion matrix and the normalized confusion matrix, which display correct and incorrect predictions across each class.

### 2.8 Statistical analysis

Continuous variables are presented as mean ± standard deviation, and categorical variables as n (%). Statistical analyses were performed with Python 3.10.12 using scikit-learn and statsmodels. Pearson correlation was used to examine relationships between key features and competency grades.

### 2.9 Ethical considerations

The study followed the Declaration of Helsinki. All data were de-identified. Video recordings were stored on encrypted servers accessible only to authorized researchers.

## 3 Results

### 3.1 Characteristics of the study subjects

A total of 114 mother-newborn dyads were included in the final analysis. Baseline maternal, neonatal, and obstetric characteristics are summarized in Tables 2, 3.

**Table 2:** Statistical description of continuous variables.

Parameter	Mean ± SD
Neonatal weight (g)	3185.40 ± 312.00
Neonatal length (cm)	49.80 ± 1.29
Gestational age (weeks)	39.57 ± 0.87
Duration of first stage (h)	9.50 ± 4.54
Duration of second stage (h)	0.91 ± 0.63
Duration of third stage (h)	0.09 ± 0.04
Total labor duration (h)	10.48 ± 4.91
Maternal age (years)	30.93 ± 2.83
Maternal height (cm)	160.73 ± 4.86
Maternal weight (kg)	67.12 ± 7.98
Oxytocin infusion duration (h)	2.01 ± 3.45
Epidural analgesia duration (h)	6.07 ± 4.26
SBP^+^ immediately postpartum (mmHg)	116.89 ± 5.89
SBP DBP^+^ immediately postpartum (mmHg)	70.35 ± 5.00
^a^SBP at 2 h postpartum (mmHg)	116.27 ± 6.47
^b^DBP at 2 h postpartum (mmHg)	69.68 ± 4.73
Intrapartum blood loss (ml)	218.07 ± 102.58
Postpartum blood loss (2 h, ml)	24.91 ± 6.02
Time since pethidine administration (h)*	6.76 ± 8.06
Time to first nutritive suckling (min)	52.26 ± 15.77

Time interval from last administration of pethidine-promethazine combination to successful fetal delivery; ^a^SBP, Systolic Blood Pressure; ^b^DBP, Diastolic Blood Pressure. ^*^Full name: the interval between the last dose of pethidine + promethazine and the successful delivery of the fetus.

**Table 3:** Frequency distributions of categorical variables (*n* = 114).

Variable	Category	n	%
Newborn sex	Male	65	57.02
	Female	49	42.98
Mode of delivery	Vertex vaginal delivery	112	98.25
	Forceps-assisted delivery	2	1.75
Maternal education level	High school / technical secondary school	5	4.39
	Associate degree (college)	17	14.91
	Bachelor’s degree	70	61.40
	Master’s degree or above	22	19.30
Abnormal fetal heart rate	Yes	91	79.82
	No	23	20.18
Gravidity	1	66	57.89
	2	27	23.68
	≥3	21	18.42
Parity	1	83	72.81
	≥2	31	27.19
Number of abortions	0	84	73.68
	1	16	14.04
	≥2	14	12.28
Nipple height/projection	Inverted	2	1.75
	Flat	11	9.65
	Short	13	11.40
	Protruding	88	77.19
High-risk factors affecting breast crawl	0	15	13.16
	1	29	25.44
	2	27	23.68
	3	32	28.07
	≥4	11	9.65
Older primipara	Yes	5	4.39
	No	109	95.61
Uterine scar	Yes	4	3.51
	No	110	96.49
Gestational diabetes mellitus (GDM)	Yes	18	15.79
	No	96	84.21
Gestational thrombocytopenia	Yes	1	0.88
	No	113	99.12
Intrahepatic cholestasis of pregnancy (ICP)	Yes	1	0.88
	No	113	99.12
Premature rupture of membranes (PROM)	Yes	37	32.46
	No	77	67.54
Placental adhesion / retained products of conception (RPOC)	Yes	10	8.77
	No	104	91.23
Postpartum hemorrhage (PPH)	Yes	3	2.63
	No	111	97.37
Obesity	Yes	4	3.51
	No	110	96.49
Chronic hypertension	Yes	1	0.88
	No	113	99.12
Hepatitis B	Yes	2	1.75
	No	112	98.25
Anemia	General anemia	5	4.39
	Thalassemia	5	4.39
	No	104	91.22
IVF (*in vitro* fertilization)	Yes	8	7.02
	No	106	92.98
Thyroid disease	Yes	28	24.56
	No	86	75.44
Breast disease	Yes	3	2.63
	No	111	97.37
Maternal fever	Yes	23	20.18
	No	91	79.82
Maternal renal pelvis separation / hydronephrosis	Yes	3	2.63
	No	111	97.37
Fetal renal pelvis separation	Yes	1	0.88
	No	113	99.12
Meconium-stained amniotic fluid	Yes	1	0.88
	No	113	99.12
High myopia	Yes	4	3.51
	No	110	96.49
Antiphospholipid antibody positivity	Yes	6	5.26
	No	108	94.74
Precipitate labor	Yes	3	2.63
	No	111	97.37
Streptococcus colonization	Yes	6	5.26
	No	108	94.74
Battledore placenta	Yes	4	3.51
	No	110	96.49
Number of pethidine + promethazine administrations	0	68	59.65
	1	39	34.21
	≥2	7	6.14
Number of phloroglucinol administrations	0	109	95.61
	≥1	5	4.39
Diazepam	Yes	2	1.75
	No	112	98.25
Nifedipine	Yes	7	6.14
	No	107	93.86
Carboprost tromethamine (Hemabate)	Yes	6	5.26
	No	108	94.74
Tranexamic acid	Yes	3	2.63
	No	111	97.37
Ergometrine	Yes	27	23.68
	No	87	76.32
Dexamethasone	Yes	1	0.88
	No	113	99.12
Neostigmine	Yes	3	2.63
	No	111	97.37
Oxytocin	Yes	51	44.74
	No	63	55.26
Location of delivery and breast crawl	LDR*	53	46.49
	Delivery room (transferred after breastfeeding)	40	35.09
	Delivery room (transferred before breastfeeding)	21	18.42

*LDR, labor-delivery-recovery room.

Mean maternal age was 30.93 ± 2.83 years, and mean gestational age was 39.57 ± 0.87 weeks; 57.02% of newborns were male. Mean total duration of labor was 10.48 ± 4.91 h, and mean intrapartum blood loss was 218.07 ± 102.58 mL. Mean time to first successful suckling was 52.26 ± 15.77 min. Breast crawl grades 0–4 accounted for 12.28%, 28.07%, 2.63%, 37.72%, and 19.30% of the sample, respectively. The breast crawl was completed in the labor-delivery-recovery (LDR) room in 46.49% of cases and in the delivery room in 35.09%; after breastfeeding, mothers and infants were transferred to the antenatal ward for observation.

### 3.2 Breast crawl competency distribution

The distribution of breast crawl competency grades across the cohort is shown in Table 4.

**Table 4:** Distribution of breast crawl competency grades.

Item	Frequency	Percentage (%)
Breast crawl grade		
0	14	12.28
1	32	28.07
2	3	2.63
3	43	37.72
4	22	19.30

For model development, the dataset was randomly split into a training/validation set (80%, *n* = 94) and a held-out test set (20%, *n* = 20), with stratification to preserve the original grade distribution as much as possible (Table 5). Class distributions across all subsets are summarized in Table 6. Due to the extremely limited number of Grade 2 cases, balanced sample representation in the test set was challenging to achieve.

**Table 5:** Mapping of the five-level competency grade onto the Widström nine stages and the endpoints of prior studies.

Grade	Widström stage reached	Endpoint in prior literature
0	Stages 1–8 completed (reaches suckling, Stage 8)	Successful breast crawl / successful suckling
1	Reaches familiarization (Stage 7); attempts suckling (Stage 8) unsuccessfully	Reached the nipple with unsuccessful suckling
2	Reaches familiarization (Stage 7) without attempting suckling	Reached the nipple without suckling attempt
3	Reaches crawling (Stage 5) but not familiarization (Stage 7)	Failed self-locating (12)
4	Stages 1–4 only (does not reach crawling, Stage 5)	No crawling

The five-level scale maps the two boundary milestones of the Widström nine stages (5)—autonomous progression to the nipple (Stages 5–7) and successful latch with suckling (Stage 8)—onto an ordinal clinical outcome. “Failed self-locating” corresponds to the endpoint used in a recent Chinese cohort study (12).

**Table 6:** Distribution of breast crawl grades in the training and test sets.

Breast crawl grade	Grade 0	Grade1	Grade2	Grade3	Grade4	Total
Training and validation set	11	26	2	35	20	94
Test set	3	6	1	8	2	20
Total	14	32	3	43	22	114

### 3.3 Overall model performance

The 3D-CNN model exhibited reasonable discriminative performance in predicting neonatal breast crawl grades. On the independent test set (*n* = 20), the model yielded a macro-average AUC of 0.77 and a micro-average AUC of 0.81.

The normalized confusion matrix (Figures 3, 4) comprehensively visualized the model’s predictive performance. The model achieved high recall for Grade 4 (no crawling) at 1.00 and Grade 3 (crawling without nipple contact) at 0.88. These findings indicated high sensitivity (with wide confidence intervals) in identifying newborns with absent or severely compromised breast crawl ability.

**Figure 3:**
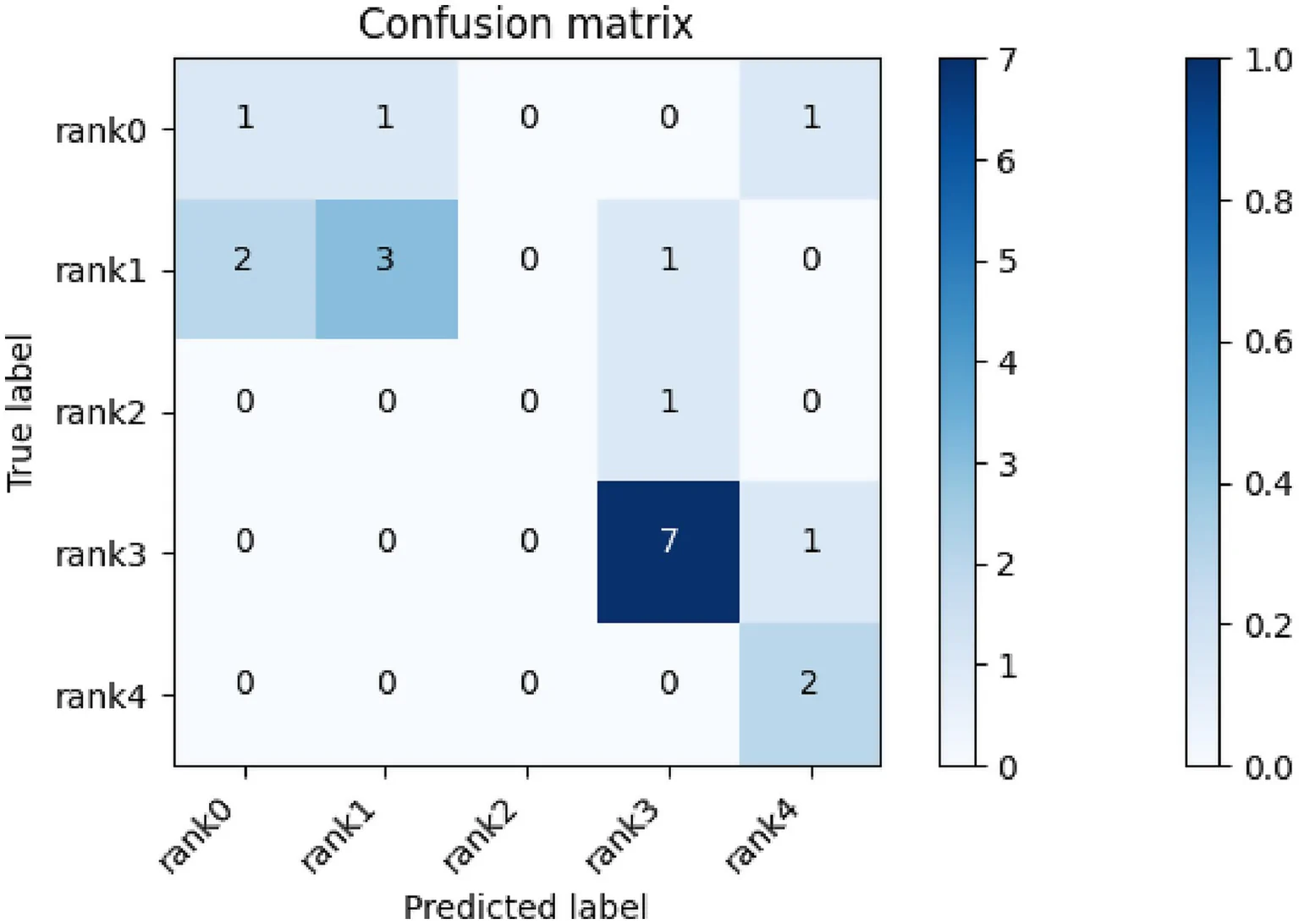
Confusion matrix for breast crawl grade prediction model.

**Figure 4:**
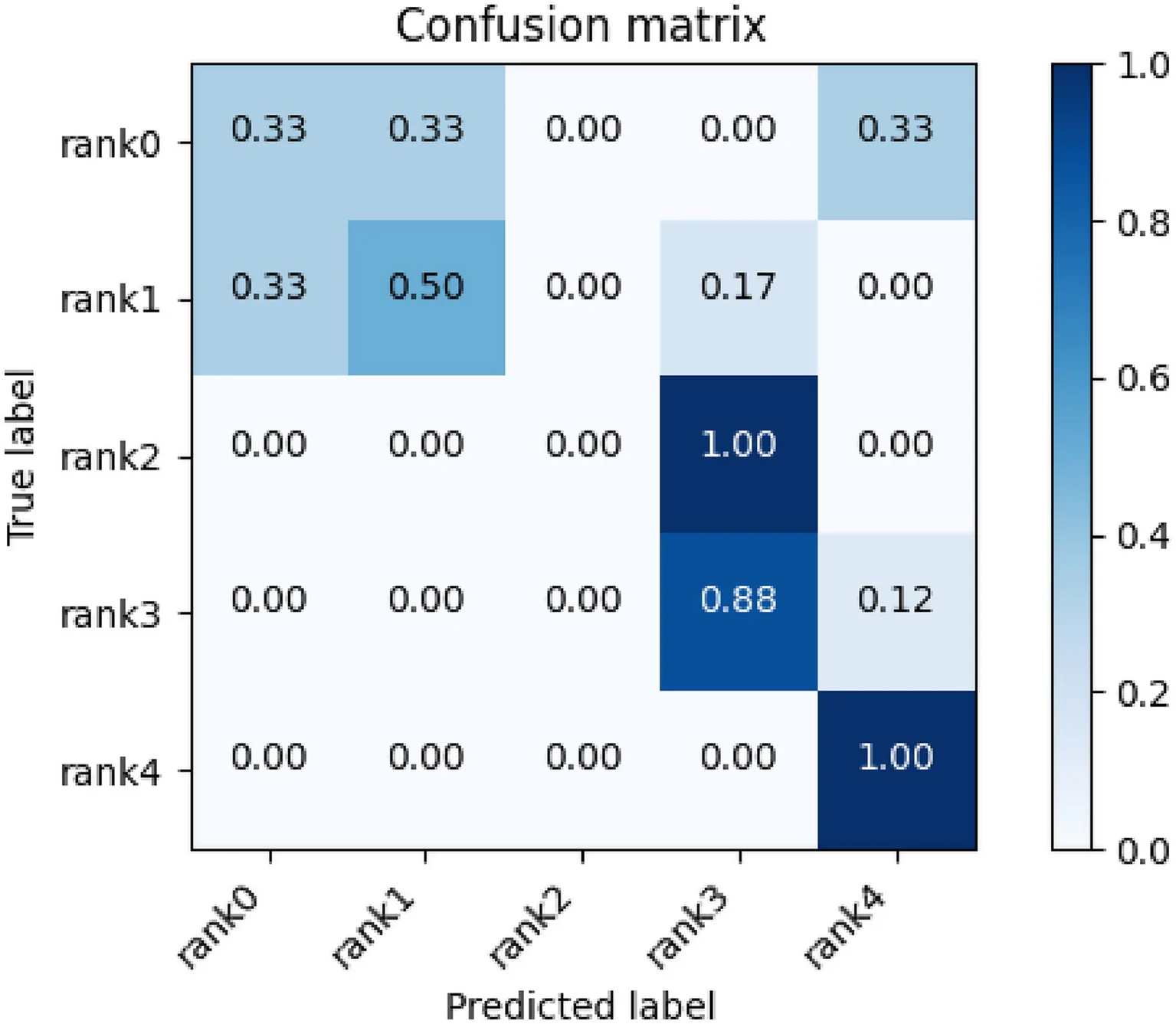
Normalized confusion matrix for breast crawl grade prediction model.

### 3.4 Grade-specific predictive performance

Detailed performance metrics for each grade are presented in Tables 7, 8. The model achieved high specificity across all grades (≥ 0.83) and overall accuracy of ≥ 0.80 for most grades, with the exception of Grade 2. Wide 95% exact Clopper-Pearson confidence intervals were observed across all metrics, which was attributable to the limited size of the test dataset. For instance, the recall value for Grade 1 was 0.50 (95% CI: 0.118–0.882).

**Table 7:** Performance evaluation of the breast crawl grade prediction model (*n* = 20).

Grade	n	Accuracy	Precision	Recall	Specificity	F1-score
Grade 0	3	0.80 (0.563–0.943)	0.33 (0.008–0.906)	0.33 (0.008–0.906)	0.88 (0.636–0.985)	0.33
Grade 1	6	0.80 (0.563–0.943)	0.75 (0.194–0.994)	0.50 (0.118–0.882)	0.93 (0.661–0.998)	0.60
Grade 2	1	0.95 (0.751–0.999)	—	0.00 (0.000–0.975)	1.00 (0.824–1.000)	—
Grade 3	8	0.85 (0.621–0.968)	0.78 (0.400–0.972)	0.88 (0.473–0.997)	0.83 (0.516–0.979)	0.82
Grade 4	2	0.90 (0.683–0.988)	0.50 (0.068–0.932)	1.00 (0.158–1.000)	0.89 (0.653–0.986)	0.67

Precision and F1 are undefined (—) because no test case was predicted as Grade 2. Values in parentheses are exact 95% confidence intervals (Clopper–Pearson). F1-scores are reported as point estimates only, as no closed-form confidence interval exists for the F1 statistic. For Grade 2, precision and F1 are undefined (—) because no test case was predicted as Grade 2 (0/0); recall is 0.00 with a 95% CI of 0.000–0.975. *n* = number of dyads per grade in the held-out test set.

**Table 8:** *Post-hoc* binary re-analysis of the held-out test set: impaired breast crawl (Grades 3–4) vs. nipple reached (Grades 0–2).

Metric	Value	95% CI
Sensitivity	1.00 (10/10)	0.692–1.000
Specificity	0.70 (7/10)	0.348–0.933
Positive predictive value	0.77 (10/13)	0.462–0.950
Negative predictive value	1.00 (7/7)	0.590–1.000
Accuracy	0.85 (17/20)	0.621–0.968

This binary grouping was obtained by collapsing the five-class predictions of the original model; it was not produced by a separately trained binary classifier, and it inherits the same limitations (including the non-nested feature selection described in the Limitations). Confidence intervals are exact Clopper–Pearson intervals.

For the clinically critical impaired-crawl grades (Grades 3 and 4), the model achieved F1-scores of 0.82 and 0.67, respectively.

For the competent crawl grades (Grades 0 and 1), the model yielded moderate predictive performance, with F1-scores of 0.33 and 0.60, respectively. The relatively low precision of 0.33 for Grade 0 indicated that the model occasionally misclassified fully competent breast crawl cases as lower-grade outcomes.

Grade 2 represented a rare intermediate phenotype. Owing to its extremely limited sample size (total *n* = 3; test set *n* = 1), the model could not produce reliable estimates for this category. Consequently, the model achieved a recall of 0.00, while precision could not be reliably quantified.

### 3.5 ROC curves and AUC

The model exhibited moderate overall discriminative performance, with a macro-average AUC of 0.77 and a micro-average AUC of 0.81. ROC curves and corresponding AUC values of the proposed model are visualized in Figure 5.

**Figure 5:**
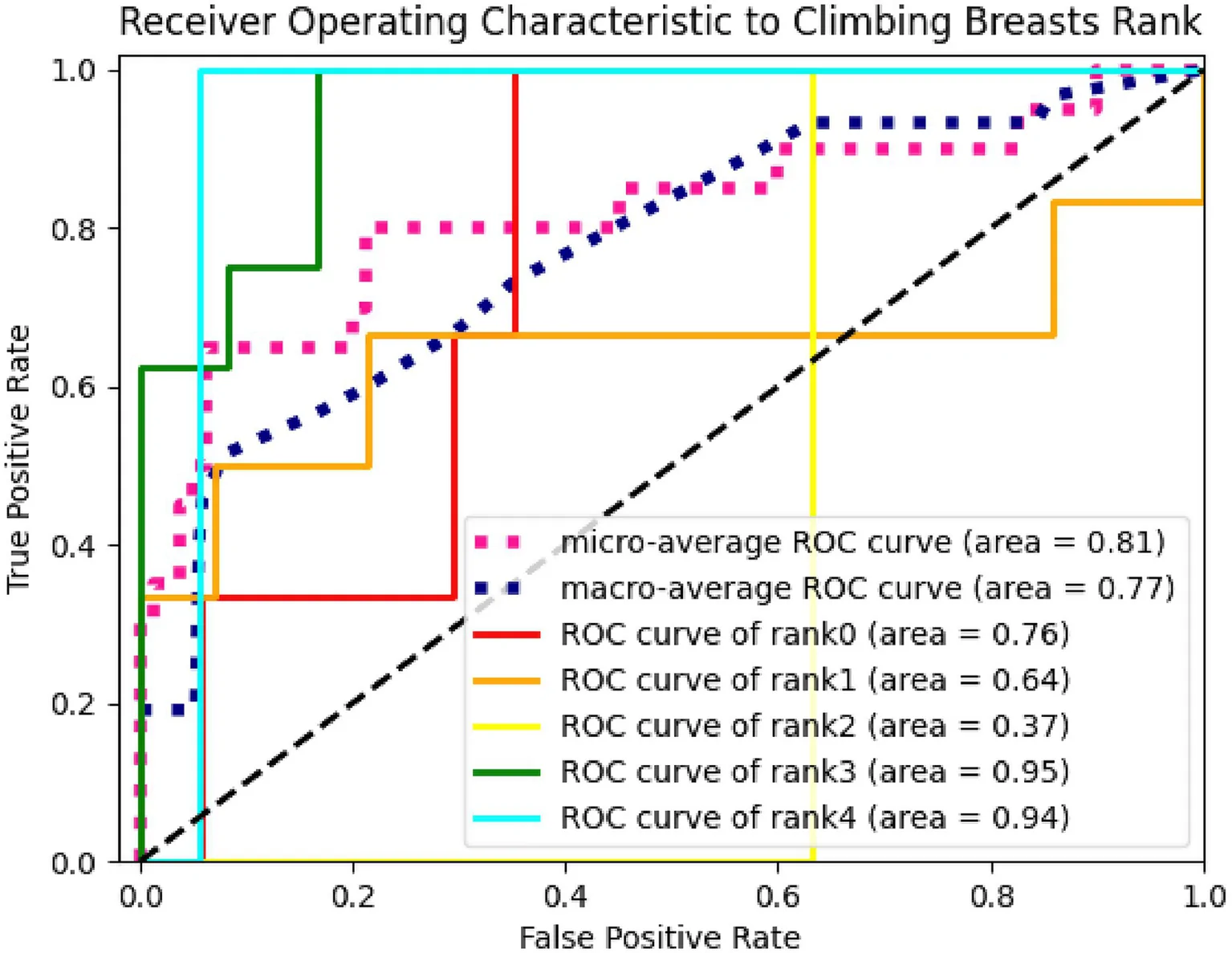
ROC curves and AUC for breast crawl grade prediction model.

### 3.6 Key predictive features identified by SHAP analysis

SHAP analysis revealed that model predictions were collectively determined by subtle behavioral sequences and specific perinatal clinical factors. The global feature importance ranking, based on mean absolute SHAP values, is shown in Figure 6.

**Figure 6:**
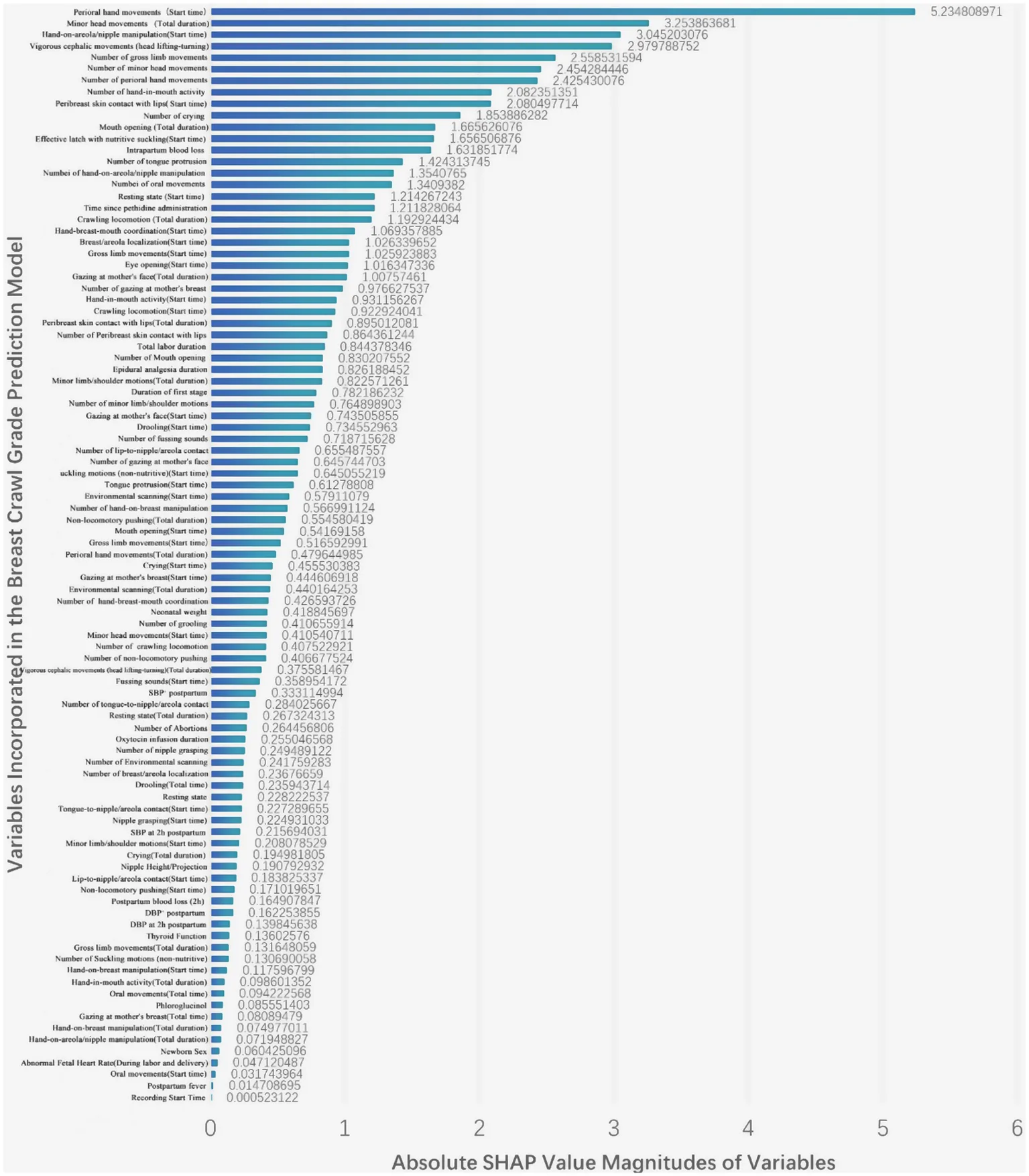
Feature composition and importance ranking of a SHAP-based breast crawl grade prediction model.

#### 3.6.1 Behavioral feature patterns associated with each grade

Grade 0 was positively associated with a higher frequency of hand-to-oral-cavity movements, a higher frequency of oral movements, an earlier onset of hand-to-perioral activity, and an earlier onset of labial skin contact.

Grade 1 was positively associated with later initiation of hand-to-areola/nipple activity, fewer hand-to-perioral movements, fewer major head movements, longer minor head movements, and fewer hand-to-oral-cavity movements.

Grade 2 was associated with earlier initiation of hand-to-perioral activity, earlier onset of labial skin contact, longer hand-breast-mouth activity, more frequent gross head movements, and fewer gazes toward the mother’s breast.

Grade 3 was positively associated with later initiation of hand-to-perioral activity, more frequent gross limb movements, longer slight limb and hand movements, fewer large head movements, and more frequent crying.

Grade 4 was positively associated with later initiation of hand-to-perioral activity, longer and more frequent slight head movements, later initiation of hand-to-areola/nipple activity, and more frequent gross limb movements.

#### 3.6.2 Influential clinical and obstetric factors

Several clinical factors were positively associated with specific grades. Labor analgesia: a shorter interval from pethidine-promethazine administration to delivery was a consistent negative predictor, associated with higher probabilities of Grades 3 and 4. Labor duration: a longer first stage of labor was associated with poorer crawling performance (Grades 3 and 4). Estimated blood loss: higher intrapartum blood loss showed variable importance across grades, but its clinical interpretation requires further investigation.

## 4 Discussion

This study developed and internally evaluated a deep learning framework for the automated, graded assessment of neonatal breast crawl competency. By integrating a 3D-CNN with SHAP interpretation, this study transcended conventional binary evaluation to establish a multidimensional, interpretable assessment framework for this complex innate neonatal behavior. Evaluated on the independent test set (*n* = 20), the model achieved moderate overall discriminative performance with a macro-average AUC of 0.77 and showed potential for identifying neonates with absent or severely impaired breast crawl (Grades 3 and 4). Notably, the relatively wide confidence intervals of all metrics were attributable to the limited test sample size. The interpretability analysis identified a set of coherent behavioral features and clinical factors that may serve as quantitative markers for the objective assessment of newborns with impaired breast crawl and may help guide individualized care for those identified as high risk. Consistent with the limitations discussed below, these findings should be regarded as a proof-of-concept demonstration rather than a validated performance estimate.

### 4.1 Implications for child health

Early breast crawl is a key component of EENC. It is estimated that full breastfeeding could prevent more than 820,000 deaths of children under 5 years each year worldwide (19). During the newborn period, breast crawl behavior is related to adequate breastfeeding and has a strong influence on nutritional status and growth. Compared with conventional manual observation, the proposed model enables objective, automated assessment of newborns at risk of suboptimal breast crawl performance. Since the model analyzes fully completed crawling episodes instead of partial early video segments, it serves as a postnatal assessment tool for the immediate postpartum period, rather than a prospective early-warning system. The model’s high sensitivity for Grade 3 (crawled but failed to reach the nipple) and Grade 4 (no crawling) supports its potential utility for standardized evaluation within the first hour after birth, though further prospective validation is still required. In clinical practice, real-time video analysis can alert clinical staff to at-risk newborns who require targeted supportive interventions, including prolonged or supervised skin-to-skin contact, reduced environmental stimulation, and timely breastfeeding guidance. This could contribute to reducing under-five mortality.

### 4.2 Decoding the behavioral signatures of proficiency and impairment

The interpretability analysis revealed associations between breast crawl performance and a series of subtle behavioral features and perinatal clinical factors. These findings validate and refine existing theory and suggest new entry points for clinical intervention. Among behavioral characteristics, the cluster of orally directed behaviors, represented by a high frequency of hand-to-oral-cavity movements, a high frequency of oral movements, and early initiation of hand-to-perioral activity, is associated with stimulation from the taste of amniotic fluid on the newborn’s hands (20). This cluster is the strongest positive predictor of good crawling performance and reflects the infant’s ability to self-soothe and regulate behavioral state (21), as well as the success of breastfeeding in full-term infants (22, 23). A low frequency of hand-to-perioral activity was associated with better performance, which may distinguish “stable tactile exploration” from “ineffective repetitive rubbing” (24, 25). This suggests that the latter may be a behavioral marker of difficulty in neonatal state regulation. An earlier onset of hand-to-areola/nipple contact indicates more purposeful and organized exploratory behavior. This action is not accidental touch but follows a sequence of orientation and then exploration (5). Its physiological significance lies in providing directional navigation for newborns and promoting maternal oxytocin secretion (10), which creates favorable physiological and behavioral conditions for successful breastfeeding establishment. Furthermore, reduced gross limb motion and abbreviated subtle head movements suggest that breast crawl quality relies less on movement magnitude and more on behavioral efficiency and functional effectiveness. An optimal crawling performance presents a smooth progression from preliminary weak movements to stable, goal-oriented crawling (14), free of redundant or prolonged motions, such as excessive arm swinging and repetitive trivial head rotation. Collectively, these behavioral features demonstrate that successful breast crawl represents a highly organized, goal-directed, and self-regulated innate behavioral pattern in newborns.

### 4.3 The influence of perinatal clinical factors

Perinatal medical intervention timing and dosage were strongly correlated with neonatal breast crawl outcomes. Specifically, a shorter interval between pethidine administration and delivery, as well as prolonged analgesic pump utilization, was linked to suboptimal breast crawl performance (26, 27). These findings indicate that intrapartum analgesics may suppress spontaneous breastfeeding-related behaviors, highlighting the necessity of precise and individualized analgesia timing management. Future studies are warranted to further explore the influences of diverse medication types on neonatal breast crawl competence. In addition, the association between a shorter first stage of labor and better performance (28, 29) highlights the importance of viewing the mother and newborn as a closely connected unit. It suggests that the mother’s birth experience and stress levels may affect the newborn’s breast crawl performance through the intrauterine environment or the quality of early postpartum interaction.

### 4.4 Limitations

This study has several limitations. Three of them affect the validity of our performance estimates and were identified during re-examination of the analysis pipeline. First, feature selection was not nested within the evaluation procedure: SHAP values were computed across the complete dataset, including test cases, before the final feature set was chosen, so the reported metrics are optimistically biased. Second, the action recognition stage was partitioned at the level of action clips rather than participants, so clips from the same neonate appeared in multiple subsets. Third, the recordings used to train the action recognition model were not excluded from the competency evaluation set. The metrics in Table 7 should therefore be read as an upper bound on achievable performance rather than as an unbiased estimate.

Annotation was performed by two researchers working jointly to consensus, and the remaining recordings were annotated by a single researcher. This approach did not generate independent duplicate annotations, so inter-rater reliability cannot be reported and systematic bias in identifying subtle behaviors cannot be excluded. We also did not separately evaluate action recognition accuracy, and the annotated data were highly imbalanced, ranging from 3,263 instances for slight head movement to 7 for locating the breast or areola. The behaviors most relevant to grading are therefore likely those recognized least reliably.

Performance was estimated on a single test set of 20 dyads, and the exact binomial confidence intervals are correspondingly wide. Grade 2 comprised only three dyads and could not be meaningfully evaluated. Because the original recordings and intermediate feature files are no longer available, we could not re-estimate performance using participant-level nested cross-validation. For the same reason, we could not compute precision–recall (PR) curves or the area under the PR curve (AUPRC), which are more informative than ROC analysis under class imbalance; moreover, at this sample size (*n* = 20) and with a rare class (a single Grade 2 case in the test set), AUPRC would be highly unstable and difficult to interpret. The model also received 140 candidate features from 114 dyads, a ratio that increases the risk of overfitting.

Two design features warrant caution. Recording ended at first suckling, so observation windows varied in length and were related to competency; frequency and duration features may therefore partly encode observation duration. In addition, midwives stimulated the rooting reflex after 45 min in neonates unable to suckle spontaneously, so the observed sequence was not entirely spontaneous in all dyads.

Finally, all participants were recruited from a single center and a demographically homogeneous population, which limits generalizability. SHAP attributions describe associations rather than causal effects, so the clinical factors identified should be treated as hypotheses rather than modifiable targets.

This work should therefore be regarded as a proof-of-concept demonstration rather than a validated prediction model, and it requires confirmation in an independent cohort before any clinical use.

### 4.5 Future directions

These limitations point to three clear priorities for future research. First, multicenter external validation with a prespecified, fully nested analytical framework is essential to generate unbiased and generalizable performance estimates; this framework should incorporate precision–recall analysis (AUPRC), which is more informative than ROC analysis under class imbalance. Second, the annotation protocol should incorporate independent duplicate coding to enable quantitative evaluation of inter-rater reliability. Additionally, the action recognition model requires comprehensive per-class evaluation, supplemented by targeted annotation of rare yet clinically critical behavioral samples. Third, systematic benchmarking against multiple comparative models is warranted, including clinical-variable-only models, models trained on manually engineered behavioral features, and alternative video-based network architectures. Further ablation analysis of the four input modalities can clarify the individual contribution of each component to the final prediction performance. Fourth, integrating additional data such as neonatal heart rate and oxygen saturation may support a more comprehensive multimodal assessment. Finally, if performance is confirmed, prospective evaluation is needed to determine whether real-time feedback to clinicians improves breastfeeding initiation, which is the outcome that ultimately matters.

## Data Availability

The original contributions presented in the study are included in the article/Supplementary material, further inquiries can be directed to the corresponding author.
