# Supplementary material for: A deep learning model for automated assessment of neonates at risk of impaired breast crawl: a proof-of-concept video-based study
Source: Front Public Health. 2026 Oct 23;14:1908206. doi: 10.3389/fpubh.2026.1908206 (PMC13739771; doi:10.3389/fpubh.2026.1908206)
Supplement: Supplementary file 1 [file Data_Sheet_1.pdf]

Supplementary Materials

Table S1: Breast Crawl Competency Grading Scale

Grade	Meaning	Endpoint in prior literature
0	Neonate autonomously crawls to nipple, latches, and achieves nutritive suckling	Successful breast crawl / successful suckling
1	Neonate crawls to nipple and attempts latching/suckling but is unsuccessful	Reached the nipple with unsuccessful suckling
2	Neonate crawls to nipple but does not attempt latching/suckling	Reached the nipple without suckling attempt
3	Crawling movements are observed, but neonate does not reach nipple autonomously	Failed self-locating [12]
4	No crawling movements are observed.	No crawling

Table S2. Distribution of annotated action instances across behaviour classes.

**Behaviour category**	**Validation set**	**Training set**	**Training and validation set**	**Test set**	**Total**
Crying	36	328	364	90	454
Eye opening	5	46	51	12	63
Looking around	184	1656	1840	460	2300
Slight head movement	261	2350	2611	652	3263
Oral movements	239	2152	2391	597	2988
Mouth opening	38	343	381	95	476

Slight hand, limb and shoulder movement	14	124	138	34	172
Gross limb movement	8	70	78	19	97
Pushing without displacement	20	180	200	50	250
Hand-to-perioral activity	19	169	188	46	234
Tongue protrusion	19	169	188	46	234
Drooling	2	17	19	4	23
Hand-to-areola/nipple activity	18	160	178	44	222
Hand-to-breast activity	19	173	192	47	239
Looking at the mother's breast	6	58	64	16	80
Looking at the mother's face	4	36	40	9	49
Cooing	10	85	95	23	118
Head lifting/turning (gross head movement)	37	332	369	92	461
Lips touching the skin around the	1	11	12	2	14

breast					
Crawling	8	68	76	18	94
Mouthing and sucking movements	12	104	116	29	145
Locating the mother's breast/areola	1	5	6	1	7
Hand-to-breast-to-mouth activity	4	35	39	9	48
Hand-to-oral cavity	3	25	28	7	35
Tongue touching the nipple/areola	2	22	24	6	30
Lips touching the nipple/areola	1	12	13	3	16
Holding the nipple	1	11	12	3	15
Correct latch and suckling	2	20	22	5	27
Continuous correct sucking	2	16	18	4	22
Total	976	8777	9753	2423	12176

Notes: Instances were obtained by manual annotation of 19 recordings (12,176 in total) and randomly partitioned into training (70%, n = 8,777), validation (10%, n = 976) and test (20%, n = 2,423) subsets. The distribution is highly imbalanced, ranging from 3,263 instances for slight head movement to 7 for locating the mother's breast/areola.

Table S3. Behavioural descriptors included in the model for each behaviour.

Behaviour	Onset time	Duration	Frequency	Behaviour	Onset time	Duration	Frequency
Crying	1	1	1	Looking at the mother's breast	1	1	1
Relaxation	0	0	0	Looking at the mother's face	1	1	1
Eye opening	1	0	0	Cooing	1	0	1
Looking around	1	1	1	Head lifting/turning (gross head movement)	1	1	1
Slight head movement	1	1	1	Lips touching the skin around the breast	1	1	1
Oral movements	1	1	1	Crawling	1	1	1
Mouth opening	1	1	1	Rest	1	1	1
Slight hand, limb and shoulder movement	1	1	1	Mouthing and sucking movements	1	0	1

Gross limb movement	1	1	1	Hand-to-breast-to-mouth activity	1	0	1
Pushing without displacement	1	1	1	Hand-to-oral cavity	1	1	1
Hand-to-perioral activity	1	1	1	Locating the mother's breast/areola	1	0	1
Tongue protrusion	1	0	1	Tongue touching the nipple/areola	1	0	1
Drooling	1	1	1	Lips touching the nipple/areola	1	0	1
Hand-to-areola/nipple activity	1	1	1	Holding the nipple	1	0	1
Hand-to-breast activity	1	1	1	Correct latch and suckling	1	0	0

Note: 1 = descriptor included in the model; 0 = descriptor not included.
