# Supplementary material for: A deep learning model for automated assessment of neonates at risk of impaired breast crawl: a proof-of-concept video-based study
Source: Front Public Health. 2026 Oct 23;14:1908206. doi: 10.3389/fpubh.2026.1908206 (PMC13739771; doi:10.3389/fpubh.2026.1908206)
Supplement: Supplementary file 2 [file Data_Sheet_2.pdf]

Supplementary Table S4.

Complete list of maternal, neonatal, and obstetric variables (n = 65) included as candidate predictors in the breast crawl competency prediction model.

No.	Variable	Type	Unit / Categories
1	Neonatal weight	Continuous	g
2	Neonatal length	Continuous	cm
3	Gestational age	Continuous	weeks
4	Duration of first stage	Continuous	h
5	Duration of second stage	Continuous	h
6	Duration of third stage	Continuous	h
7	Total labor duration	Continuous	h
8	Maternal age	Continuous	years
9	Maternal height	Continuous	cm
10	Maternal weight	Continuous	kg
11	Oxytocin infusion duration	Continuous	h
12	Epidural analgesia duration	Continuous	h
13	Systolic blood pressure (SBP) immediately postpartum	Continuous	mmHg
14	Diastolic blood pressure (DBP) immediately postpartum	Continuous	mmHg
15	SBP at 2 h postpartum	Continuous	mmHg
16	DBP at 2 h postpartum	Continuous	mmHg
17	Intrapartum blood loss	Continuous	ml
18	Postpartum blood loss (2 h)	Continuous	ml
19	Time since pethidine administration*	Continuous	h
20	Time to first nutritive suckling	Continuous	min
21	Recording start time	Continuous	clock time
22	Newborn sex	Categorical	Male / Female
23	Mode of delivery	Categorical	Vertex vaginal delivery / Forceps-assisted delivery
24	Maternal education level	Categorical	High school / Associate degree / Bachelor's degree / Master's degree or above
25	Abnormal fetal heart rate	Categorical	Yes / No
26	Gravidity	Categorical	1 / 2 / ≥3
27	Parity	Categorical	1 / ≥2
28	Number of abortions	Categorical	0 / 1 / ≥2
29	Nipple height/projection	Categorical	Inverted / Flat / Short / Protruding
30	High-risk factors affecting breast crawl	Categorical	0 / 1 / 2 / 3 / ≥4
31	Elderly primipara	Categorical	Yes / No
32	Uterine scar	Categorical	Yes / No
33	Gestational diabetes mellitus (GDM)	Categorical	Yes / No
34	Gestational thrombocytopenia	Categorical	Yes / No
35	Intrahepatic cholestasis of pregnancy (ICP)	Categorical	Yes / No
36	Premature rupture of	Categorical	Yes / No

	membranes (PROM)		
37	Placental adhesion / retained products of conception (RPOC)	Categorical	Yes / No
38	Postpartum hemorrhage (PPH)	Categorical	Yes / No
39	Obesity	Categorical	Yes / No
40	Chronic hypertension	Categorical	Yes / No
41	Hepatitis B	Categorical	Yes / No
42	Anemia	Categorical	General anemia / Thalassemia / No
43	In vitro fertilization (IVF)	Categorical	Yes / No
44	Thyroid disease	Categorical	Yes / No
45	Breast disease	Categorical	Yes / No
46	Maternal fever	Categorical	Yes / No
47	Maternal renal pelvis separation / hydronephrosis	Categorical	Yes / No
48	Fetal renal pelvis separation	Categorical	Yes / No
49	Meconium-stained amniotic fluid	Categorical	Yes / No
50	High myopia	Categorical	Yes / No
51	Antiphospholipid antibody positivity	Categorical	Yes / No
52	Precipitate labor	Categorical	Yes / No
53	Streptococcus colonization	Categorical	Yes / No
54	Battledore placenta	Categorical	Yes / No
55	Number of pethidine + promethazine administrations	Categorical	0 / 1 / ≥2
56	Number of phloroglucinol administrations	Categorical	0 / ≥1
57	Diazepam	Categorical	Yes / No
58	Nifedipine	Categorical	Yes / No
59	Carboprost tromethamine (Hemabate)	Categorical	Yes / No
60	Tranexamic acid	Categorical	Yes / No
61	Ergometrine	Categorical	Yes / No
62	Dexamethasone	Categorical	Yes / No
63	Neostigmine	Categorical	Yes / No
64	Oxytocin	Categorical	Yes / No
65	Location of delivery and breast crawl	Categorical	LDR / Delivery room (transferred after breastfeeding) / Delivery room (transferred before breastfeeding)

*SBP, systolic blood pressure; DBP, diastolic blood pressure; GDM, gestational diabetes mellitus; ICP, intrahepatic cholestasis of pregnancy; PROM, premature rupture of membranes; RPOC, retained products of conception; PPH, postpartum hemorrhage; IVF, in vitro fertilization; LDR, labor-delivery-recovery room. *Time interval from the last administration of the pethidine–promethazine combination to successful fetal delivery.*
